# Rapid Microfluidic Biosensor for Point-of-Care Determination of Rheumatoid Arthritis via Anti-Cyclic Citrullinated Peptide Antibody Detection

**DOI:** 10.3390/bios14110545

**Published:** 2024-11-10

**Authors:** Wei-Yu Tai, To-Lin Chen, Hsing-Meng Wang, Lung-Ming Fu

**Affiliations:** 1 Department of Emergency Medicine, Kaohsiung Chang Gung Memorial Hospital, Kaohsiung 83301, Taiwan; weiyutai3@cgmh.org.tw; 2 Department of Engineering Science, National Cheng Kung University, Tainan 70101, Taiwan; n98134049@gs.ncku.edu.tw (T.-L.C.); n96104496@gs.ncku.edu.tw (H.-M.W.)

**Keywords:** microfluidics, microchip, ELISA, RA, anti-CCP antibody

## Abstract

Rheumatoid arthritis (RA) is a chronic autoimmune disorder that causes extensive damage to multiple organs and tissues and has no known cure. This study introduces a microfluidic detection platform that combines a microfluidic reaction chip with a micro-spectrometer to accurately detect the anti-cyclic citrullinated peptide antibody (anti-CCP Ab) biomarker, commonly associated with arthritis. The surface of the microfluidic reaction chip is functionalized using streptavidin to enable the subsequent immobilization of biotinylated-labeled cyclic citrullinated peptide (biotin–CCP) molecules through a streptavidin–biotin reaction. The modified chip is then exposed to anti-CCP Ab, second antibody conjugated with horseradish peroxidase (HRP) (2nd Ab-HRP), 3,3′,5,5′-tetramethylbenzidine (TMB), and a stop solution. Finally, the concentration of the anti-CCP Ab biomarker is determined by analyzing the optical density (OD) of the colorimetric reaction product at 450 nm using a micro-spectrometer. The detection platform demonstrated a strong correlation (R^2^ = 0.9966) between OD and anti-CCP Ab concentration. This was based on seven control samples with anti-CCP Ab concentrations ranging from 0.625 to 100 ng/mL. Moreover, for 30 artificial serum samples with unknown anti-CCP Ab concentrations, the biosensor achieves a correlation coefficient of (R^2^ = 0.9650). The proposed microfluidic detection platform offers a fast and effective method for accurately identifying and quantifying the anti-CCP Ab biomarker. Thus, it offers a valuable tool for the early diagnosis and monitoring of RA and its progression in point-of-care settings.

## 1. Introduction

Rheumatoid arthritis (RA) is a chronic autoimmune disease marked by joint inflammation and tissue damage. While its exact cause remains unknown, RA involves a combination of genetic and environmental factors, making early and effective diagnosis essential to prevent irreversible damage [1,2]. The diagnosis of RA involves a combination of joint damage assessment, serological testing, and evaluation using standardized scales in medical examinations [3]. Therefore, an early diagnostic method that is both sensitive and specific is crucial for enabling timely intervention and preventing irreversible damage. This underscores the vital importance of advanced biological detection techniques in the diagnosis of RA [4].

Rheumatoid factor (RF) and tumor necrosis factor-alpha (TNF-α) [5] are commonly used biomarkers in RA diagnosis; however, both lack the specificity needed to distinguish RA from other inflammatory diseases accurately. RF is often present in other autoimmune conditions, while TNF-α, though significant in RA-related inflammation, is also elevated in various inflammatory diseases, limiting its diagnostic utility for RA. In contrast, anti-cyclic citrullinated peptide antibody (anti-CCP Ab) is particular to RA, making it a valuable marker for precise diagnosis, particularly in the early stages of the disease [6,7,8]. Furthermore, most RA patients have detectable levels of anti-CCP antibodies, specifically in the immunoglobulin G (IgG) class. Consequently, anti-CCP Ab has gradually replaced RF and TNF-α as a more reliable risk factor for diagnosing RA. The anti-CCP Ab test provides a quantitative measurement result, and a reading of less than 20 IU/mL is often considered a reliable early detector of RA. Despite advancements in diagnostic technology, existing RA detection methods still present challenges, particularly in point-of-care (POC) applications.

Current RA diagnostic methods, including electrochemical immunosensors [9,10], particle-enhanced turbidimetric immunoassays (PETIAs) [11], magnetic particles [12], and nanoplasmonic sensing chips [13], provide valuable insights but face limitations for point-of-care (POC) applications [14,15,16,17,18,19]. Electrochemical immunosensors require specialized electrodes and controlled conditions, while PETIAs depend on complex laboratory equipment, making both less suitable for rapid, accessible testing. Magnetic nanoparticle-based assays, though effective, involve multistep processes that increase operational complexity, and nanoplasmonic sensing chips often require precise optical setups, further limiting their practicality in POC settings. In response, we developed a novel microfluidic platform tailored for POC applications, specifically for early RA detection.

Microfluidic detection technology has advanced significantly in recent years, enabling a variety of detection methods across diverse applications [20,21,22,23,24,25,26]. A key development has been the integration of microfluidic platforms with enzyme-linked immunoassays (ELISAs), which utilize enzymatic reactions to quantify antigens or antibodies [27,28,29]. In indirect ELISAs, capture agents such as peptides, antigens, or aptamers, often biotinylated for improved immobilization [30,31,32], are commonly used. For detecting anti-CCP antibodies, the ELISA employs biotin–CCP as the capture antigen, followed by a secondary antibody conjugated with horseradish peroxidase (HRP). This enzyme catalyzes a reaction with 3,3′,5,5′-tetramethylbenzidine (TMB) and hydrogen peroxide (H_2_O_2_), enabling quantitative determination of anti-CCP Ab concentration. Studies have shown that streptavidin can enhance the immobilization efficiency of biotin–CCP by binding to its biotin moiety [33,34]. Additionally, microfluidic devices have proven essential for facilitating rapid and efficient analysis [35,36,37,38,39,40]. Consequently, microfluidic-based ELISA systems have become promising tools for enhancing the sensitivity, speed, and reliability of RA biomarker detection. Overall, this microfluidic-based detection system holds significant potential for enhancing RA diagnostic accessibility and precision in clinical and point-of-care settings.

**Figure 1:**
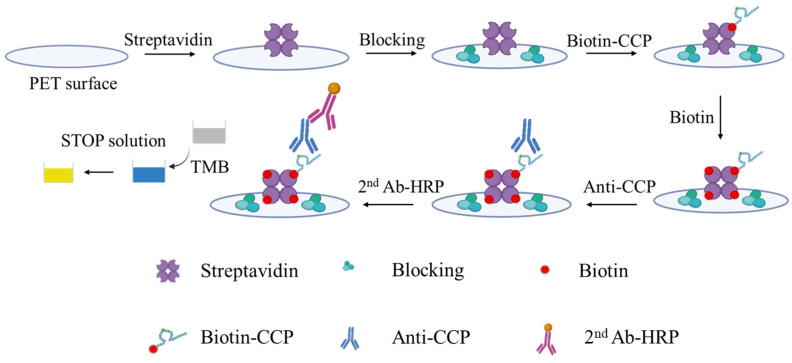
Schematic of proposed modified indirect ELISA detection method.

Accordingly, the present study proposes a novel microfluidic platform for the POC detection of anti-CCP Ab, consisting mainly of a poly (methyl methacrylate) (PMMA) reaction chip, a micro-spectrometer, a detection platform with a hot plate, and a Raspberry Pi with a touch screen. The surface of the reaction chip is functionalized using streptavidin to immobilize biotin–CCP molecules through a streptavidin–biotin interaction [34,41]. The unoccupied binding sites on the detection substrate are then blocked using bovine serum albumin (BSA) to minimize nonspecific binding. In the detection process, the detection region of the chip is exposed to anti-CCP Ab, followed by a secondary antibody conjugated with horseradish peroxidase (HRP) (2nd Ab-HRP) to facilitate signal amplification. The subsequent catalytic reaction between HRP and 3,3′,5,5′-tetramethylbenzidine (TMB) is analyzed using the micro-spectrometer at 450 nm, with the resulting optical density (OD) signal being used to quantify the anti-CCP Ab concentration. The proposed modified indirect ELISA detection method is shown schematically in Figure 1.

The practicality of the proposed detection system is demonstrated by measuring the anti-CCP Ab concentrations in 30 artificial serum RA samples and comparing the results with those obtained using a conventional spectroscopic method. The two sets of results show good agreement (R^2^ = 0.9650), thereby confirming the accuracy and stability of the proposed detection method. This accuracy underscores the potential of this detection system for early RA diagnosis in POC settings.

## 2. Materials and Methods

### 2.1. Reagents

The reagents used in this study included streptavidin (5 mg/mL) that was bought from ChemScene (Monmouth Junction, NJ, USA), which was used to coat the chip surface for stable biotinylated molecule immobilization. Biotin–CCP (1 mg/mL) (peptide sequence: Biotin-HQCHQEST-Cit-GRSRGRCGRSGS-COOH), which contains a disulfide bond between the Cyc3 and Cyc16 residues, was obtained from FineTest Inc. (Wuhan, China) and served as the capture antigen for selective detection of anti-CCP antibodies. The anti-cyclic citrullinated peptide antibody (100 ng/mL = 30 IU/mL), sample diluent, and antibody diluent, as well as the 2nd antibody conjugate HRP (2nd Ab-HRP), were sourced from FineTest Inc. Additionally, 1% bovine serum albumin (BSA), diluted from a 10% stock solution was used as a blocking agent to reduce nonspecific binding, with phosphate-buffered saline (137 mM sodium chloride, 2.7 mM potassium chloride, 8.1 mM sodium hydrogen phosphate, 1.5 mM potassium dihydrogen phosphate), a wash buffer, and PBS containing 0.5% Tween 20 (PBST) used for rinsing steps. A substrate solution containing a mixture of TMB and H_2_O_2_ (in a 1:1 ratio) and a stop solution (1 N sulfuric acid) were obtained from R&D Systems (Minneapolis, MN, USA). Biotin was acquired from Sigma-Aldrich (Burlington, MA, USA). All reagents were stored according to the manufacturer’s instructions to maintain stability and effectiveness.

### 2.2. Fabrication Details

**Figure 2:**
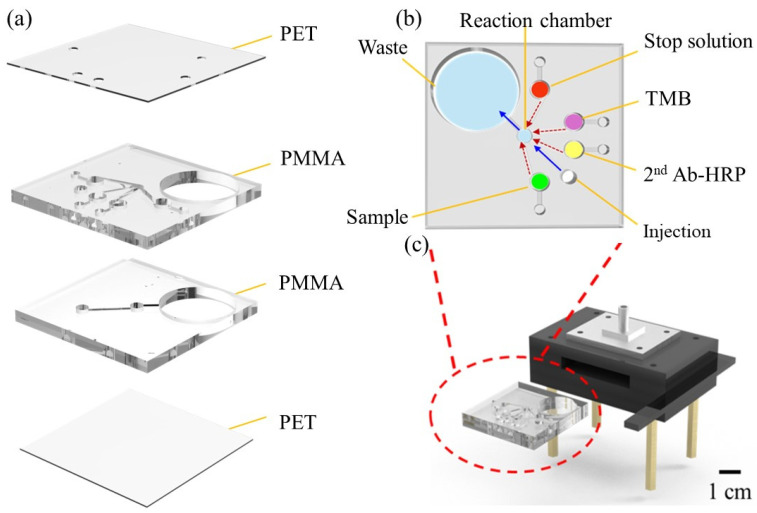
Schematic illustrations of proposed microfluidic RA detection platform: (**a**) assembly breakdown of microchip, (**b**) top view of microchip, and (**c**) insertion of microchip into detection platform.

Figure 2a,b present an exploded view and a top view of the proposed microfluidic reaction chip, respectively. In Figure 2b, the flow directions for the reagents are illustrated. The red arrows indicate the movement of the reagents from the top layer to the reaction chamber in the bottom layer, driven by syringe pressure. Once the reagents enter the bottom layer and the reaction is complete, they are pushed from the reaction chamber to the waste chamber, as indicated by the blue arrows, also using syringe pressure. Figure 2c shows the insertion of the microchip into the detection system. As shown in Figure 2a, the microchip comprised two patterned PMMA substrates, each with a thickness of 3 mm, and two PET cover layers, each with a thickness of 0.2 mm. Each of the four layers measured 40 × 40 mm^2^. Figure 2b shows the detailed configuration of the upper PMMA layer, in which the injection and reaction chambers had diameters of 3 mm, the reagent chambers had diameters of 2.2 mm, and the reservoirs had diameters of 4 mm, respectively. The waste chamber, with a diameter of 18 mm, served to collect all the waste reagents produced during the preparation and reaction processes. All the chambers and reservoirs were connected by microchannels with a width and depth of 1 × 1 mm^2^.

The substrates and upper cover layer were designed using SolidWorks (2018) and patterned by a CO_2_ laser (C180II, GCC, New Taipei City, Taiwan). The process of patterning was thoroughly explained in previous studies [42,43]. The PET/PMMA/PMMA/PET layers were accurately aligned and sealed using a hot-press bonding technique. Initially, the chip was subjected to gentle, constant pressure while the temperature gradually raised to 105 °C over 10 min. The pressure was then increased to 10 kg/cm^2^ and maintained for an additional 10 min, with the temperature kept constant. Finally, the chip was allowed to cool to room temperature (25 °C) for 14 min.

### 2.3. Optical Analysis System and Microchip Reaction Process

**Figure 3:**
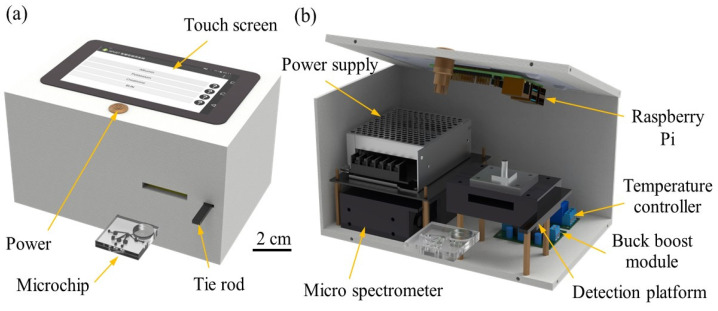
Schematic illustrations of proposed optical analysis system: (**a**) side view of optical analysis system and (**b**) arrangement of main components in optical analysis system.

Figure 3a,b show the optical analysis system and the basic arrangement of its main components. A comprehensive description of the system and its operation can be found in previous studies [23]. Therefore, these details are not repeated here. In short, the main components of the system included a micro-spectrometer (OTO, Hsinchu City, Taiwan), a detection platform, an LED light source, a 450 nm filter, Raspberry Pi with a touchscreen, and a temperature controller. The filter removed unwanted wavelengths from the LED light source, improving the accuracy and reliability of the optical detection system. The filtered light then traveled through the reaction zone before being transmitted to the micro-spectrometer via a fiber optic cable. This cable was securely attached to an aluminum alloy base using an SMA (SubMiniature version A) connector from Fusoh Shoji Co., Ltd in Tokyo, Japan. Meanwhile, the temperature controller maintained a constant temperature during the ELISA reaction process and ensured that the enzymatic reactions proceeded efficiently.

### 2.4. Experimental Details

For the reagent preparation, streptavidin stock powder (5 mg) was dissolved in 1 mL of DI water and then further diluted with DI water to 0.5 mg/mL. A 1% BSA solution was prepared by diluting a 10% BSA stock solution with DI water to 1% BSA. The capture antigen, biotin–CCP, was prepared by dissolving 1 mg of lyophilized biotin–CCP powder in 1 mL of PBS to 1 mg/mL, followed by further dilution with PBS to 2 μg/mL. The biotin solution was prepared by dissolving biotin in PBS to 2 mg/mL and then further diluting with PBS to 2 μg/mL. A standard solution of anti-CCP lyophilized powder at 500 μg/mL was prepared by dissolving 500 μg of anti-CCP antibody in sample diluent, then serially diluted to achieve control samples with anti-CCP concentrations of 1.57, 3.125, 6.25, 12.5, 25, 50 and 100 ng/mL. Finally, a stock solution of detection antibody conjugated with HRP (2nd Ab-HRP) was diluted 400-fold with antibody diluent to the working concentration.

The PET/PMMA microfluidic chip was initially coated with streptavidin, then sealed to prevent evaporation and incubated overnight at 4 °C to ensure stable binding. After washing with PBST to remove unbound streptavidin, a 1% BSA solution was applied for two hours at 25 °C to block nonspecific binding, followed by another PBST wash. The chip was subsequently incubated overnight at 4 °C with 2 μg/mL biotin–CCP in PBS, washed, and then treated with a 2 μg/mL biotin solution for two hours at 25 °C to conjugate any remaining streptavidin sites. After a final wash, all reagents except for the anti-CCP samples were added to the chambers in the second layer, and the chip was sealed for use.

In the detection process, 20 μL of a sample containing the anti-CCP antibody biomarker was injected into the reaction chamber using a syringe and incubated for 30 min. After a washing step, 2nd Ab-HRP was injected into the chip under syringe pressure and incubated for an additional 30 min. Following another rinse to remove any unbound secondary antibodies, tetramethylbenzidine (TMB) was injected into the chip and incubated for 10 min to facilitate the colorimetric reaction. Finally, a stop solution was injected to terminate the reaction, and the chip was analyzed using an optical detection system to determine the concentration of anti-CCP antibodies.

## 3. Results and Discussions

### 3.1. Specificity of Detection Process

**Figure 4:**
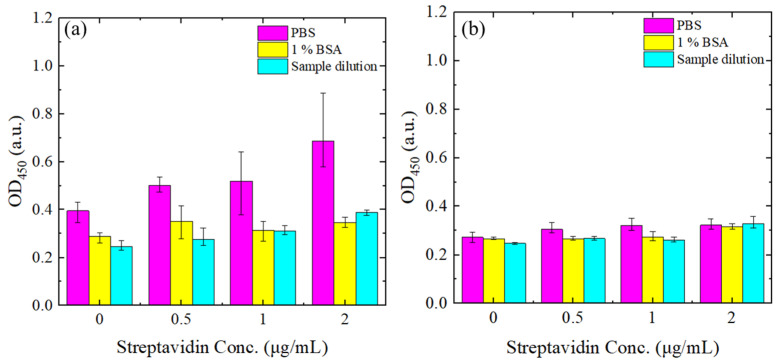
OD at 450 nm for different blank samples and streptavidin concentrations in the range of 0–2 μg/mL. (**a**) Without biotin addition. (**b**) With biotin addition. Error bars represent the range, calculated as the difference between the maximum and average values across three independent measurements.

In the detection process proposed in this study, we initially functionalized the detection surface with streptavidin to reduce nonspecific binding and enhance the binding efficiency of biotin–CCP molecules. To evaluate the specificity of this functionalized surface, we tested three different blocking buffers: PBS, 1% BSA, and a sample dilution buffer. Each sample was treated with 2 μg/mL of biotin, along with varying concentrations of streptavidin (0–2 μg/mL). The OD results were then compared for samples both with and without biotin, as illustrated in Figure 4a,b.

In Figure 4a, it is observed that when PBS was used as the blocking reagent, the OD increased in direct proportion to the concentration of streptavidin. In contrast, both 1% BSA and the sample dilution buffer resulted in lower OD values compared to PBS; however, the OD still increased with the concentration of streptavidin. Figure 4b demonstrates that adding biotin significantly reduced the OD across all buffers, regardless of the type of blocking buffer employed. Based on these results, an optimal streptavidin concentration of 0.5 μg/mL was chosen to minimize non-specific binding and enhance capture efficiency.

### 3.2. Parameter Optimization of Anti-CCP Ab Immunoassay

The proposed detection method employed a two-step blocking process consisting of the use of a blocking agent, followed by the application of biotin–CCP. The aim of the two-step process was to improve the performance of the modified ELISA method by minimizing the background noise, thereby achieving a more accurate and reliable detection of the anti-CCP Ab analyte. To determine the optimal conditions for the modified ELISA detection method, experiments were conducted to examine the optimal blocking agent and biotin–CCP concentration, respectively.

**Figure 5:**
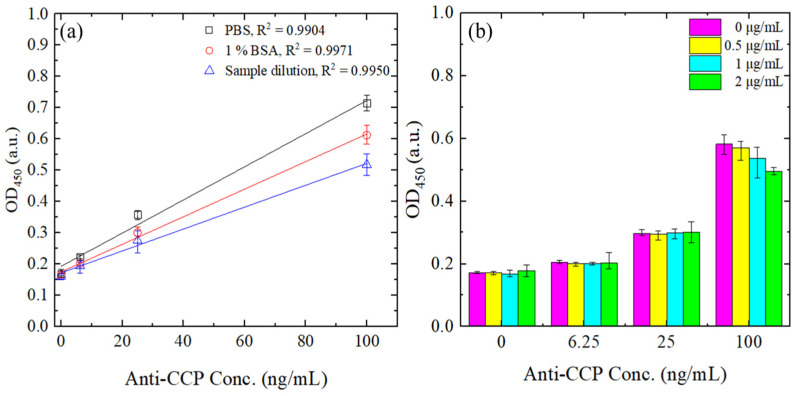
(**a**) Effects of different blocking buffers on OD value for different anti-CCP concentrations, and (**b**) effects of different biotin–CCP concentrations on OD value for different anti-CCP Ab concentrations. Error bars represent the range, calculated as the difference between the maximum and average values across three independent measurements.

Figure 5a shows the change in OD with anti-CCP Ab concentrations from 0 to 100 ng/mL, using the three different blocking buffers as mentioned in Section 3.1. Note that the anti-CCP Ab concentration range covers the typical detection range for RA diagnosis [44,45]. As shown, the 1% BSA and sample dilution buffers both blocked the substrate, as indicated by the lower OD signal intensity for all values of the anti-CCP concentration. However, the 1% BSA buffer resulted in an improved correlation between the OD intensity and the anti-CCP Ab concentration (R^2^ = 0.9971). Thus, 1% BSA buffer was selected as the optimal buffer for the detection process.

Figure 5b shows the OD values obtained for different anti-CCP Ab concentrations when utilizing biotin buffers with concentrations ranging from 0 to 2 μg/mL. At low concentrations of anti-CCP Ab, the two-step blocking method only had a negligible effect on the OD intensity. However, as the anti-CCP Ab concentration increased to 100 ng/mL, the OD value also increased. In other words, the effectiveness of the two-step blocking method in suppressing background noise was particularly pronounced at higher anti-CCP Ab concentrations. Among the four biotin–CCP concentrations considered, the highest concentration of 2 μg/mL resulted in the most significant reduction in the background noise. Thus, the biotin concentration was selected as 2 μg/mL in all the remaining experiments.

**Figure 6:**
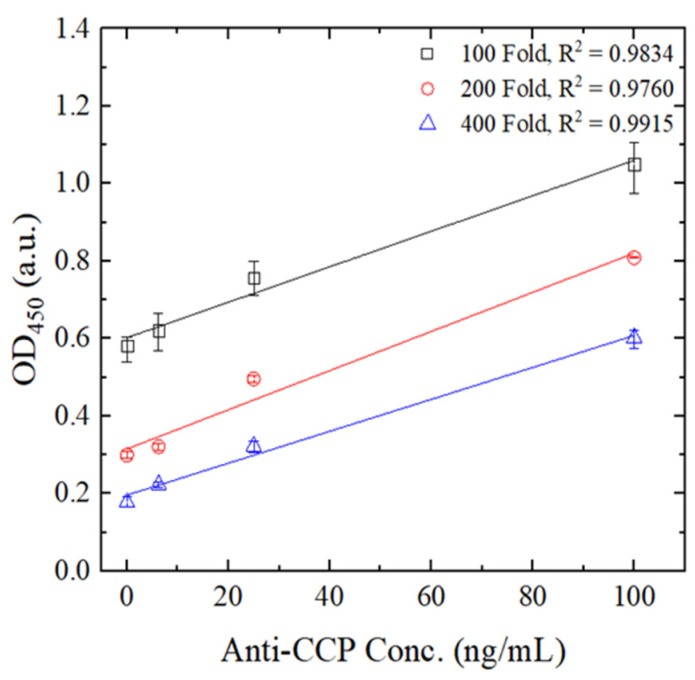
Variation in OD intensity with anti-CCP Ab concentration for 2nd Ab-HRP reagents with different dilution factors. Error bars represent the range, calculated as the difference between the maximum and average values across three independent measurements.

A series of experiments was conducted to determine the optimal dilution factor for the second Ab-HRP to enhance signal amplification while minimizing non-specific binding. The results shown in Figure 6 indicate that the OD signal intensity increased as the dilution factor decreased, demonstrating stronger signal responses at lower dilution factors. However, at lower dilution factors, such as a 100-fold dilution, there was a significant increase in non-specific binding, which could compromise the specificity of the assay. As the dilution factor increased, the correlation coefficient (R^2^) between OD intensity and anti-CCP concentration improved, which indicates better linearity and detection accuracy. Therefore, a 400-fold dilution factor was selected to optimize both signal amplification and assay specificity for the remaining experiments.

### 3.3. Calibration Curve

**Figure 7:**
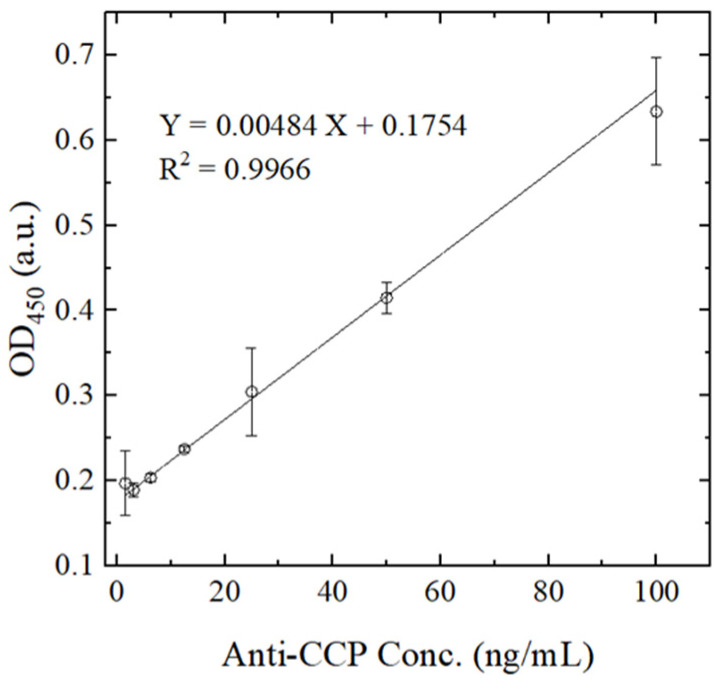
Calibration curve for anti-CCP Ab concentration determination at wavelength of 450 nm. Error bars represent the range, calculated as the difference between the maximum and average values across three independent measurements.

Figure 7 shows the OD values obtained by the proposed microfluidic detection platform for seven anti-CCP Ab control samples with known concentrations of 1.57, 3.125, 6.25, 12.5, 25, 50 and 100 ng/mL. For each sample, detection was carried out under the optimal conditions as described in the previous section. The calibration curve was tested three times for each sample to ensure accuracy and reproducibility. The OD intensity and sample concentration demonstrate a strong correlation (R^2^ = 0.9966). Therefore, the basic stability and reliability of the detection system are confirmed. Moreover, the limit of detection (LOD) of the proposed microfluidic ELISA system is 1.57 ng/mL.

### 3.4. Detection Results for Samples with Unknown Anti-CCP Ab Concentrations

**Figure 8:**
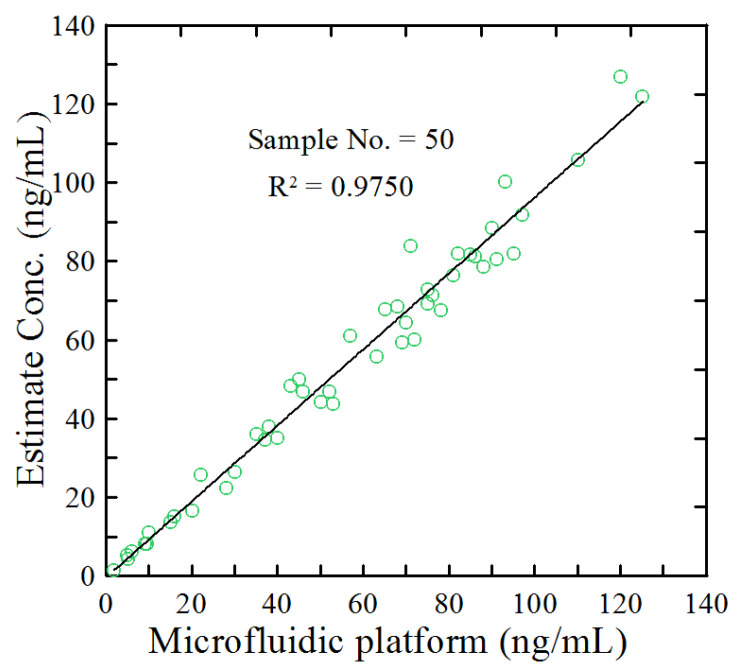
Comparison of detection results and measurement results for 50 water-based samples with unknown anti-CCP Ab concentrations.

The detection performance of the proposed platform was further evaluated using 50 water-based samples with unknown anti-CCP Ab concentrations. Figure 8 compares the detection results with those obtained from measurements using the Absorbance 96 plate reader (Enzo Life Sciences, Farmingdale, NY, USA). The two sets of results show excellent agreement, indicating the reliability of the proposed detection system, with a linear regression coefficient of R^2^ = 0.9750. Moreover, the recovery rate for 50 samples was 96.1%, with a standard deviation of 5.1% in the measured RA concentrations. This finding confirms the robust and reliable performance of the proposed system when used for unknown samples and suggests its potential for accurate anti-CCP Ab concentration measurements.

**Figure 9:**
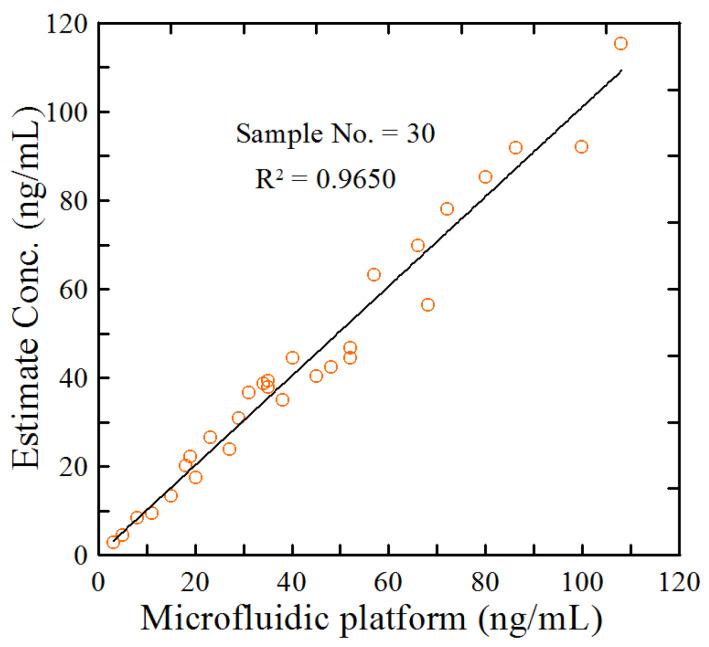
Comparison of detection results and measurement results for 30 artificial serum samples with unknown anti-CCP Ab concentrations.

The practical feasibility of the proposed detection system was evaluated by determining the concentrations of 30 artificial serum samples with unknown anti-CCP concentrations. The corresponding results are shown in Figure 9. A good agreement is once again observed between the detection results and the experimental measurements (R^2^ = 0.9650), and the recovery and standard deviation of the 30 samples are 95.6% and 5.5%, respectively. In Figure 9, a comparison is shown between the detection results obtained from the proposed system and those from the Absorbance 96 plate reader (Enzo Life Sciences, USA). The strong correlation between the two sets of results demonstrates the effectiveness of the proposed system in detecting anti-CCP antibodies in real-world serum samples. Table 1 provides a detailed comparison of existing studies on the determination of anti-CCP antibodies, alongside several previously reported methods from the literature. This table emphasizes the differences between these methods and our proposed ELISA-based microfluidic system. As a result, the suitability of the current microfluidic ELISA detection platform, with its modified detection process, for anti-CCP detection has been confirmed.

## 4. Conclusions

This study has presented a novel microfluidic platform for the detection of the anti-CCP Ab biomarker linked to RA using a modified indirect ELISA method. The proposed system comprises a micro-heater, a micro-spectrometer, a detection platform, a Raspberry Pi with a touch screen, an LED light source, and a 450 nm filter. The ELISA process has been tailored for use on a microfluidic chip and incorporates multiple pre-processing steps aimed at improving the detection performance, including sample incubation with second Ab-HRP, TMB, and a stop solution. In the proposed method, the concentration of the anti-CCP Ab biomarker is determined from the measured OD value of the colorimetric reaction product using a calibration curve and custom-developed OD conversion software (V).

The experimental results showed a strong correlation (R^2^ = 0.9966) between the OD values and anti-CCP antibody concentrations in seven control samples with known concentrations ranging from 1.57 to 100 ng/mL. Furthermore, the analysis of 30 artificial serum samples with unknown anti-CCP antibody concentrations resulted in a correlation coefficient of R^2^ = 0.9650. Thus, the results confirm that the proposed platform has significant promise for POC testing and monitoring of patients with RA.

## Figures and Tables

**Table 1:** Comparison of analytical methods in anti-CCP assay.

Method	Analysis Time	Device Price	Detection Range	LOD	Instrument Type	Ref.
Electrochemical	>2 h	High	10–1000 IU/mL	2.5 IU/mL	Benchtop	[7]
Electrochemical	>60 min	High	1/2–1/64 serum dilution	N/A	Benchtop	[9]
Photoelectric	>2 h	High	1/100 serum dilution	N/A	Benchtop	[10]
Particle-enhanced turbidimetric immunoassay (PETIA)	>3 h	High	13–430 U/mL	12 U/mL	Benchtop	[11]
Magnetic particle	12 min	High	0.7–2000 U/mL	0.7 U/mL	Benchtop	[12]
Nanoplasmonic sensing	12 min	Low	N/A	N/A	Handheld	[13]
Electrochemical	2 h	High	0.125–2000 pg/mL	0.0125 pg/mL	Benchtop	[46]
Electrochemical	40 min	High	1–800 IU/mL	0.82 IU/mL	Benchtop	[47]
Magnetic particle	55 min	High	0.2–59 U/mL	N/A	Benchtop	[48]
Proposed method	<70 min	Low	1.57–100 ng/mL	1.57 ng/mL	Handheld	This work

N/A: not applicable.

## Data Availability

The data presented in this study are available upon request from the corresponding author.

## References

[1] Zhu J.N., Dai L., Li Y.H., Guo X.Y., Zhang X.M. (2019). Meta-analysis: Compared with anti-CCP and rheumatoid factor, could anti-MCV be the next biomarker in the rheumatoid arthritis classification criteria?. Clin. Chem. Lab. Med..

[2] Zhang M., Huang M., Chen W., Li Y., Xu L., Zhou F., Zhang H., Chen W. (2022). Oxygen supplementation liposomes for rheumatoid arthritis treatment via synergistic phototherapy and repolarization of M1-to-M2 macrophages. Chem. Eng. J..

[3] Smith J., Johnson L. (2022). Importance of Early Detection in Rheumatoid Arthritis. Innovation.

[4] Brown A., Davis M. (2022). Advances in RA Biomarker Diagnostics. Innovation.

[5] Zhang M., Zhang S., Tang Y., Zhuang X., Yang X., Guo Y. (2023). Targeted therapy for autoimmune diseases based on multifunctional frame nucleic acid system: Blocking TNF-α-NF-κB signaling and mediating macrophage polarization. Chem. Eng. J..

[6] Schellekens G.A., Visser H., de Jong B.A., van den Hoogen F.H., Hazes J.M., van de Putte L.B., van Venrooij W.J. (2000). The diagnostic properties of rheumatoid arthritis antibodies recognizing a cyclic citrullinated peptide. Arthritis Rheum..

[7] Guerrero S., Castillo J., Fuentes M., Cruz M., Marican A., Meza N., Cortez C., Gabler C. (2020). Electrochemical biosensor for the simultaneous determination of rheumatoid factor and anti-cyclic citrullinated peptide antibodies in human serum. Analyst.

[8] Gao S., Huang M., Chen W., Zhang F., Zhang H., Tan C., Zhang J. (2022). A biolayer interferometry-based, aptamer–antibody receptor pair biosensor for real-time, sensitive, and specific detection of the disease biomarker TNF-α. Chem. Eng. J..

[9] Lin C.Y., Yu S.Y., Lee G.J., Chen Y.J., Chen Y.C., Huang C.H. (2022). Peptide-based electrochemical sensor with nanogold enhancement for detecting rheumatoid arthritis. Talanta.

[10] Chen H.-M., Tsai Y.-H., Hsu C.-Y., Wang Y.-Y., Hsieh C.-E., Chen J.-H., Chang Y.-S., Lin C.-Y. (2023). Peptide-Coated Bacteriorhodopsin-Based Photoelectric Biosensor for Detecting Rheumatoid Arthritis. Biosensors.

[11] Fu L., Zou Y., Li Y., Zhou P., Zhang W., Ma Z. (2015). A modified quick PETIA for detecting anti-CCP antibodies in human serum. Anal. Methods.

[12] Ramos K.C., Macías M.D.P.C. (2021). Microdevice immunoassay with conjugated magnetic nanoparticles for rapid anti-cyclic citrullinated peptide (anti-CCP) detection. Talanta.

[13] Dutta P., Nam H.W., Kim K.S., Min J. (2022). Combining portable solar-powered centrifuge to nanoplasmonic sensing chip with smartphone reader for rheumatoid arthritis detection. Chem. Eng. J..

[14] Mumtaz Z., Rashid Z., Ali A., Arif A., Ameen F., AlTami M.S., Yousaf M.Z. (2023). Prospects of microfluidic technology in nucleic acid detection approaches. Biosensors.

[15] Huang R., Quan J., Su B., Cai C., Cai S., Chen Y., Mou Z., Zhou P., Ma D., Cui X. (2022). A two-step competition assay for visual, sensitive and quantitative C-reactive protein detection in low-cost microfluidic particle accumulators. Sens. Actuators B Chem..

[16] Qian M., Zeng Y., Li M., Gao Q., Zhang C., Qi H. (2024). Electrogenerated Chemiluminescence Biosensor for Quantization of Matrix Metalloproteinase-3 in Serum via Target-Induced Cleavage of Oligopeptide. Biosensors.

[17] Bakuova N., Toktarkan S., Dyussembinov D., Azhibek D., Rakhymzhanov A., Kostas K., Kulsharova G. (2023). Design, simulation, and evaluation of polymer-based microfluidic devices via computational fluid dynamics and cell culture “on-chip”. Biosensors.

[18] Li F., You M., Li S., Hu J., Liu C., Gong Y., Yang H., Xu F. (2020). based point-of-care immunoassays: Recent advances and emerging trends. Biotechnol. Adv..

[19] Flora F.C., Relvas S.B., Silva F.A., Freire M.G., Chu V., Conde J.P. (2023). Combined Use of Ionic Liquid-Based Aqueous Biphasic Systems and Microfluidic Devices for the Detection of Prostate-Specific Antigen. Biosensors.

[20] Bartosh A.V., Sotnikov D.V., Zherdev A.V., Dzantiev B.B. (2023). Handling Detection Limits of Multiplex Lateral Flow Immunoassay by Choosing the Order of Binding Zones. Micromachines.

[21] Nguyen H.V., Yang J., Van Nguyen H., Poo H., Seo T.S. (2023). Development of a high-throughput centrifugal microsystem for enzyme-linked immunosorbent assay to detect SARS-CoV-2. Chem. Eng. J..

[22] Ko C.H., Tseng C.C., Lu S.Y., Lee C.C., Kim S., Fu L.M. (2025). Handheld microfluidic multiple detection device for concurrent blood urea nitrogen and creatinine ratio determination using colorimetric approach. Sens. Actuators B Chem..

[23] Chen S.J., Lu S.Y., Tseng C.C., Huang K.H., Chen T.L., Fu L.M. (2024). Rapid Microfluidic Immuno-Biosensor Detection System for the Point-of-Care Determination of High-Sensitivity Urinary C-Reactive Protein. Biosensors.

[24] Ko C.H., Liu C.C., Huang K.H., Fu L.M. (2023). Finger Pump Mcrofluidic Detection System for Methylparaben Detection in Foods. Food Chem..

[25] Hiniduma K., Bhalerao K.S., De Silva P.I.T., Chen T., Rusling J.F. (2023). Design and Fabrication of a 3D-Printed Microfluidic Immunoarray for Ultrasensitive Multiplexed Protein Detection. Micromachines.

[26] Akhtar A.S., Soares R.R., Pinto I.F., Russom A. (2023). A portable and low-cost centrifugal microfluidic platform for multiplexed colorimetric detection of protein biomarkers. Anal. Chim. Acta.

[27] Khan M., Zhao B., Wu W., Zhao M., Bi Y., Hu Q. (2023). Distance-based microfluidic assays for instrument-free visual point-of-care testing. TrAC Trends Anal. Chem..

[28] Chen F., Hu Q., Li H., Xie Y., Xiu L., Zhang Y., Guo X., Yin K. (2023). Multiplex detection of infectious diseases on microfluidic platforms. Biosensors.

[29] Balzer A.H., Whitehurst C.B. (2023). An analysis of the biotin–(strept) avidin system in immunoassays: Interference and mitigation strategies. Curr. Issues Mol. Biol..

[30] Sanders A., Gama R., Ashby H., Mohammed P. (2021). Biotin immunoassay interference: A UK-based prevalence study. Ann. Clin. Biochem..

[31] Wang X.M., Li S., Li L.H., Song J.X., Lu Y.H., Zhou Z.W., Zhang L. (2022). Triple quantitative detection of three inflammatory biomarkers with a biotin-streptavidin-phycoerythrin based lateral flow immunoassay. Anal. Biochem..

[32] Tang F., Wang Y., Wang D., Yang Y., Chang J., Sun H., He J. (2024). Streptavidin-biotin system-mediated immobilization of a bivalent nanobody onto magnetosomes for separation and analysis of 3-phenoxybenzoic acid in urine. Anal. Methods.

[33] Zhang J., Zhou T., Wang Z., Xu W., Ding S., Ge J. (2009). DNA-directed immobilisation of glycomimetics for glycoarrays application: Comparison with covalent immobilisation, and development of an on-chip IC50 measurement assay. Biosens. Bioelectron..

[34] Berth M., Willaert S., De Ridder C. (2018). Anti-streptavidin IgG antibody interference in anti-cyclic citrullinated peptide (CCP) IgG antibody assays is a rare but important cause of false-positive anti-CCP results. Clin. Chem. Lab. Med..

[35] Arias-Alpízar K., Sánchez-Cano A., Prat-Trunas J., Sulleiro E., Bosch-Nicolau P., Salvador F., Oliveira I., Molina I., Sánchez-Montalvá A., Baldrich E. (2022). Magnetic Bead Handling Using a Paper-Based Device for Quantitative Point-of-Care Testing. Biosensors.

[36] Burgos-Flórez F., Rodríguez A., Cervera E., De Ávila M., Sanjuán M., Villalba P.J. (2022). Microfluidic Paper-Based Blood Plasma Separation Device as a Potential Tool for Timely Detection of Protein Biomarkers. Micromachines.

[37] Abbas N., Song S., Chang M.-S., Chun M.-S. (2023). Point-of-Care Diagnostic Devices for Detection of *Escherichia coli* O157:H7 Using Microfluidic Systems: A Focused Review. Biosensors.

[38] Mitrogiannopoulou A.-M., Tselepi V., Ellinas K. (2023). Polymeric and Paper-Based Lab-on-a-Chip Devices in Food Safety: A Review. Micromachines.

[39] Mishra R., Alam R., McAuley D., Bharaj T., Chung D., Kinahan D.J., Nwankire C., Anderson K.S., Ducrée J. (2022). Solvent Selective Membrane Routing and Microfluidic Architecture Towards Centrifugal Automation of Customisable Bead Based Immunoassays. Sens. Actuators B Chem..

[40] Neumair J., Kröger M., Stütz E., Jerin C., Chaker A.M., Schmidt-Weber C.B., Seidel M. (2023). Flow-Based CL-SMIA for the Quantification of Protein Biomarkers from Nasal Secretions in Comparison with Sandwich ELISA. Biosensors.

[41] Boozer C., Ladd J., Chen S., Jiang S. (2006). DNA-directed protein immobilization for simultaneous detection of multiple analytes by surface plasmon resonance biosensor. Anal. Chem..

[42] Hong T.F., Ju W.J., Wu M.C., Tai C.H., Tsai C.H., Fu L.M. (2010). Rapid prototyping of PMMA microfluidic chips utilizing a CO_2_ laser. Microfluid. Nanofluid..

[43] Wu Y.T., Yang C.E., Ko C.H., Wang Y.N., Liu C.C., Fu L.M. (2020). Microfluidic detection platform with integrated micro-spectrometer system. Chem. Eng. J..

[44] Rönnelid J., Turesson C., Kastbom A. (2021). Autoantibodies in rheumatoid arthritis–laboratory and clinical perspectives. Front. Immunol..

[45] Khudhair H.A.A. (2023). A study of the roles of some immunological biomarkers in the diagnosis of rheumatoid arthritis. J. Med. Life.

[46] Ma J., Li D., Sun B., Hou X., Zhang-Peng X., Li W., Zhang Y., Hu F., Shi X. (2022). Label-free Electrochemical Immunosensor for Sensitive Detection of Rheumatoid Arthritis Biomarker Anti-CCP-ab. Electroanalysis.

[47] Chinnadayyala S.R., Cho S. (2020). Electrochemical immunosensor for the early detection of rheumatoid arthritis biomarker: Anti-cyclic citrullinated peptide antibody in human serum based on avidin-biotin system. Sensors.

[48] Wu T.H., Tsai Y.C., Kuo F.C., Lee M.S., Hu C.C., Lee G.B. (2023). A microfluidic platform for detection and quantification of two biomarkers for rheumatoid arthritis. Sens. Actuators B Chem..

